# Energy Cost of Resistance Exercises: an Uptade

**DOI:** 10.2478/v10078-011-0056-3

**Published:** 2011-10-04

**Authors:** Victor M. Reis, Roberto S. Júnior, Adam Zajac, Diogo R. Oliveira

**Affiliations:** 1 Research Centre for Sport Sciences, Health and Human Development (CIDESD), Vila Real, Portugal; 2 Department of Sport Sciences, Exercise and Health, University of Trás-os-Montes and Alto Douro (UTAD), Vila Real, Portugal; 3 Rio de Janeiro Federal University, Physical Education Post-Graduation Program, Rio de Janeiro, Brazil; 4 Department of Sports Training, Academy of Physical Education, Katowice, Poland

**Keywords:** strength training, energy expenditure, aerobic energy, anaerobic energy

## Abstract

The use of resistance exercises and of typical strength training methods have been progressively used to control body mass and to promote fat mass loss. The difficulties involved in the energy cost calculation during strength training are associated with the large amount of exercises and their several variations. Mean values between ≈3 and 30 kcal·min^−1^ are typically reported but our studies indicate that it may attain values as high as 40 kcal·min^−1^ in exercises which involve a large body mass. Therefore, in our opinion, the next step in research must be the isolated study of each of the main resistance exercises. Since the literature is scarce and that we do consider that the majority of the studies present severe limitations, the aim of this paper is to present a critical analysis of the energy cost estimation methods and provide some insights that may help to improve knowledge on resistance exercise. It seems necessary to rely on the expired O_2_ measurements to quantify aerobic energy. However, it is warranted further attention on how this measure is performed during resistance exercises. In example, studies on the O_2_ on-kinetics at various conditions are warranted (i.e. as a function of intensity, duration and movement speed). As for anaerobic lactic energy, it is our opinion that both the accumulated oxygen deficit and the blood lactate energy equivalent deserve further studies; analyzing variations of each method as an attempt to establish which is more valid for resistance exercise. The quantification of alactic anaerobic energy should be complemented by accurate studies on the muscle mass involved in the different resistance exercises. From the above, it is concluded that knowledge on the energy cost in resistance exercises is in its early days and that much research is warranted before appropriate reference values may be proposed.

## Introduction

Physical activity by the use of resistance exercise (RE) is a common modern trend. This trend is not limited to high-performance athletes, but also in physical rehabilitation programs and in physical activity with aesthetical or health-promotion purposes. As a sign of times, the American College of Sports Medicine (ACSM, 2006) recommends the inclusion of strength training (ST) in training routines that aim the prevention, control and treatment of degenerative diseases related with sedentary lifestyles. Indeed, those training methods are progressively more and more used in exercise programs designed to address body mass control and fat mass loss. Concomitantly, the research on the acute and chronic adaptations to ST as well as to the execution of RE has increased much in the past decade or so. Additionally, research on the energy expenditure (EE) or energy cost (EC) involved in the execution of RE and in ST has also increased exponentially.

Research shows an increase in EE during and after a session of RE, although the total contribution of ST to the daily EE seems more related to its influence during exercise itself (Poehlman et al., 2002; Melanson et al., 2005). The difficulty to assess EE during ST and the large variation of results, from 2,7 and 11 kcal·min^−1^ in men (Ballor et al., 1989; Pichon et al., 1996; Melanson et al., 2002; Thornton and Potteiger, 2002; Hunter et al., 2003; Phillips and Ziuraitis, 2003, Garatachea et al., 2007; Silva et al., 2007) and from 2,3 to 5,2 kcal·min^−1^ in women (Ballor et al., 1989; Pichon et al., 1996; Binzen et al., 2001; Phillips and Ziuraitis, 2003), is related to the amount of exercises and their variations, such as: muscle groups that are elicited; type of equipment that is used; number of exercises and repetitions; load; execution time in the various movement phases; exercise order; and recovery time between sets.

The large variability of the values presented above, turns research almost obsolete in terms of being able to predict with an acceptable error which is the EE in a standard session of ST. In our opinion, the next step in ST research should be the isolated study of the main resistance exercises. Being able to identify the bioenergetics in each exercise and the way it varies with its different variations or in different populations, will improve the design of ST programs. In cases where the aim of the ST is to promote body mass loss or fat mass loss, the choice of exercises, loads, recovery periods, total training volume (and other characteristics of a training session) should be done according to the knowledge of the EC of each and every exercise that is used. Indeed, the bioenergetics of RE is little known, especially in what concerns the quantification of the EC in isolated exercises (Scott et al., 2006), and are mainly focused in squat (Garatachea et al., 2007; Robergs et al. 2007) and bench press exercise (Robergs et al. 2007; Scott et al., 2009; 2011). These rare studies usually include the quantification of the EC through the evaluation of the aerobic and anaerobic fractions of energy release during and post-exercise. For such, the physiological measures often used are oxygen uptake (VO_2_) during and post-exercise, the accumulated oxygen deficit during exercise and the blood lactate post-exercise.

Considering the scarcity of studies and the fact that we do consider that the majority of the studies present severe limitations, the aim of the present paper is to present a critical analysis of the methods that are typically used to quantify the EC and to propose some new insights suitable to improve knowledge about RE and ST.

### Energy cost vs. energy expenditure

The reader may notice that in the past section we have referred two concepts: energy cost (EC) and energy expenditure (EE). We will explain the rationale behind the separate concepts. We chose to use EE when the methods that are used allow a direct quantification and with no measurement error. The measurement error herein is limited to the technological error with the use of gold-standard equipments and techniques; that is, very low. As such, only when the aerobic fraction of energy release is assessed with VO_2_ measurement and only when the anaerobic energy involved in the exercise is negligible, it is licit to consider that the EE is truly measured. So, we have already a first and severe limitation to use this concept in ST, as RE are typically characterized by a significant anaerobic energy release. For these reasons we prefer to refer to the concept of energy cost (EC). The EC represents the total amount of energy that is necessary to perform an exercise and it includes the two fractions of energy, both aerobic and anaerobic. The aerobic fraction can be directly measured without error (other than the technological error) through the VO_2_ measurement; and the anaerobic fraction can only be estimated. The latter, being estimated, it involves necessarily a certain amount of error adding to the technological error associated with the equipments and techniques of measurement.

This separation can be considered conceptual or even merely operational. If the reader prefers, merely operational so be. We do not disregard the use of EE, but it is important to draw attention to the fact that VO_2_ measurements are only a part of the total energy demand (that we prefer to call energy cost). Moreover, it is also important to remind the reader that even VO_2_ measurements, when performed during recovery periods (between exercises or post-session) do not quantify with precision the aerobic energy release. In fact, during post-exercise periods, the VO_2_ represents several mechanisms that the human body uses to reestablish its homeostasis. Hence, post-exercise VO_2_ does not quantifies the energy demand (energy cost) of any given exercise.

Therefore, we consider the EC concept to be more precise and more suitable to be used. In the present paper, we will only use this concept from now on.

### Aerobic energy cost

Aerobic EC is usually assessed by indirect calorimetric, with the measurement of the VO_2_ content in expired gases during exercise. The respiratory exchange ratio (*R*) is the expression of the respiratory quotient in the ventilation and can also be measured in the expired gases. The *R* may serve to estimate the relative substrate oxidation (Wilmore and Costill, 2004) in the muscle cell (*R*≈1,0 for predominant carbohydrate oxidation, *R*≈0,7 for predominant fat oxidation and *R*≈0,8 for predominant protein oxidation). On the other hand, for each *R* value there is an energy equivalent by liter of O_2_ uptake. In example, the energy equivalent for *R*=0,7 is 4,69 kcal·L^−1^ O_2_; for *R*=0,8 is 4,80 kcal·L^−1^ O_2_; and for *R*=1,0 it is 5,05 kcal·L^−1^ O_2_ (Wilmore and Costil, 2004). At rest conditions, *R* is typically 0,7 to 0,8 and it may attain a value above 1,0 at severe exercise intensities (i.e. those above the lactate threshold).

The use of VO_2_ as a quantitative measure of EC and the use of *R* as an indicator of the appropriate energy equivalent assumes that gas exchanges are measured during a metabolic equilibrium state (when there is a steady-state VO_2_ in the mouth). In practical terms, this means that the assessment of the aerobic energy cost is more valid the lower the exercise intensity and the higher the exercise duration. Grossly, one may consider as valid the following conditions of exercise: i) exercise intensities below that corresponding to the lactate threshold (LT) and a duration above 3-min; ii) exercise intensities comprised between that corresponding to the LT and that corresponding to the maximal VO_2_ and a duration above 5-min (or a duration that is necessary to attain a steady-state).

In running, cycling and swimming exercise, as well as in some other typical exercise modes, VO_2_ kinetics is well described as a function of the intensity and the duration of exertion. However, this is not the case with RE. On the other hand, the intensity which corresponds to the start of an exponential and fast accumulation of lactate in the blood is also scarcely analyzed. Indeed, the lactate threshold (LT) in the blood, identified as being somewhere between 70 and 80% of maximal VO_2_ in running or cycling exercise, it is not that well established during RE. Some exploratory data found LT to be around 30% 1-RM (Barros et al., 2004; Oliveira et al., 2006) in leg press, bench press and biceps curl exercises. Rocha et al. (2010) confirmed, in a more careful study, a value of around 32% for LT in inclined leg press (45°). In addition, the maximal VO_2_ concept as a reference to establish exercise intensity cannot be used in RE until science is able to uncover at which % of 1-RM does the VO_2_ attains its maximal value in the various RE. The understanding of the full oxidative capacity of the muscles involved in each RE is necessary to point out the intensities and durations at which the VO_2_ kinetics in RE are to be studied and, subsequentely, the intensities at which the EC can be considered almost fully aerobic.

The few studies about the aerobic EC in isolated resistance exercises show that in the bench press it can be as low as 1,5 kcal·min^−1^ (mean value of men and women performing at 50% 1-RM), for a total EC of 4,7 kcal·min^−1^ (Scott et al,, 2009). Robergs et al. (2007) describe a total EC much higher in men performing bench press and ½ squat at 40 and 70% 1-RM (10 to 19 kcal·min^−1^). In the latter study it is not possible to conclude how much was the aerobic EC but it is likely to be higher compared to that reported by Scott et al. (2009).

Our preliminary results (unpublished data) with recreational resistance-trained men, show that in bench press, triceps extension and latt pull down, the total EC is almost similar and calculated to be around 3 to 5 kcal·min^−1^ at low intensities (from 12 to 24% 1-RM). In these same 3 exercises performed at 80% 1-RM, the total EC varied between 10 and 12 kcal·min^−1^, with 22 to 36% released from anaerobic sources (≈2 to 4 kcal·min^−1^). Our data suggest that at intensities below 30% 1-RM the energy may be almost fully aerobic (disregarding that from the O_2_ stores which are included in the early O_2_ deficit) and that at higher loads the CE may increase concomitantly to the increases in the anaerobic energy release (maintaining almost unchanged the aerobic energy release). This hypothesis is yet to prove and only careful analysis on the VO_2_ kinetics, may confirm the negligible anaerobic contribution at low workloads (up to 30% 1-RM). In the ½ squat we found much different estimations. The total EC at lower intensities (from 12 to 24% 1-RM) varied between 10 and 12 kcal·min^−1^, but at 80% 1-RM attained 40 kcal·min^−1^, with no more than ≈6 kcal·min^−1^ from aerobic sources (other than the O_2_ stores). The variability of the data (confidence interval of the predicted value) and the imprecision of the EC (standard error of the regression between VO_2_ and workload) were larger in this exercise, compared to those previously identified. Although we did not analyzed the VO_2_ on-kinetics with multi exponential modeling, in the ½ squat an apparent slow component was present at intensities below 30% 1-RM (as shown by a difference between end VO_2_ and 2^nd^ minute VO_2_ higher than 300 ml·min^−1^).

### Anaerobic energy cost

The methods which are typically used to assess the anaerobic EC are less precise compared to those assessing aerobic EC. A variety of indirect methods have been used, but none of them is indisputably accepted as the most accurate. The gold standard method to asses alactic and lactic anaerobic energy release would warrant muscle biopsy, thereby allowing the quantification of the energy sources inside the muscle cell (i.e. high-energy phosphates and glycogen) as well as an accurate measure of metabolite accumulation in the muscle (i.e. muscle lactate). A limitation of this technique is due to the fact that only a minor portion of the human muscle tissue may be submitted to a biopsy. Moreover, it may be necessary to obtain several samples of tissue located at different depths to attain a sample that is representative of the muscle (Gollnick et al., 1972) and that mirrors the muscle heterogeneity in terms of fiber types (Sjonstrom and Fridén, 1984). On the other hand, the fact that this procedure is highly invasive does misadvises its use.

To assess the lactic portion of anaerobic EC the most referred measure in the literature is the energy equivalent of the peak blood lactate (BL) accumulation post-exercise. Usually this measure is completed with an assumption of the alactic energy sources, with a value that may vary with exercise and that is often estimated by the temporal constant of the fast O_2_ on-response during exercise and according to di Prampero et al. (1981) up to 36.8 mlO_2_·kg^−1^. The pioneer studies by Margaria et al. (1963), later followed by Cerreteli et al. (1969) and subsequently completed by those of di Prampero (for references see di Prampero, 1981) allowed the establishment of a quantitative energy equivalent for post-exercise lactate accumulation in the blood that could be used to quantify the energy yielded by the anaerobic lactic source during running or swimming exercise (generally between 2.7 and 3.3 ml O_2_ kg^−1^·m*M*^−1^). It is a fact that di Prampero clearly stated that this equivalent does not represents an energy equivalent of lactate formation, rather representing an amount of energy that could be attributed to the lactic metabolism when the rate of lactate formation greatly surpasses that of its elimination (di Prampero, 1981; di Prampero and Ferreti, 1999). As such, the explanations provided by di Prampero (1981) allow to conclude that during sub maximal exercise intensities, especially at those when blood lactate can be sustained over time (irrespective of being below or above the typical 4 m*M* threshold), that it may be unnecessary to include such measurements to estimate the total energy cost of exercise. The rationale behind this idea is quite simple and straightforward. The blood lactate values above that of resting conditions during sub maximal exercise are probably due to an initial lactate formation (di Prampero and Ferreti, 1999). Subsequently the VO_2_ progressively attains a steady-state and is able to match the energy demand, thereby turning unnecessary to consider the initial lactate formation in the overall energy cost. Despite the various sources of error described for this method in the literature (ex. Medbø and Toska, 2001), in our opinion, the main limitation to its application in RE is that every study that has supported the BL energy equivalent was performed on running, cycling or swimming exercise. Therefore, there are no experimental data that may support the value for the BL energy equivalent in RE. In RE this method has been used mostly by Scott (Scott et al., 2006; 2009; 2011).

The alternative to use the BL energy equivalent added to the alactic sources assumptions is the accumulated oxygen deficit (AOD). This is a measure which includes the two components and that does not require invasive techniques. The AOD determination is possible from VO_2_ measurement and allows the quantification of the aerobic and anaerobic fraction of energy release in relation to the overall EC. This method, rarely used in RE (Robergs et al., 2007) has been vastly used for more than 20 years in other types of exercise such as running (Reis et al., 2004; Reis et al., 2005), cycling (Buck and McNaughton, 1999) and more recently in swimming (Reis et al., 2010^a^,^b^) and it is considered by some as the most realistic available measure of anaerobic energy release in man during high-intensity exercise (Saltin, 1990; Gastin, 1994; Nakamura and Franchini, 2006). As with many other methods and techniques which are currently used in exercise physiology, the AOD is based in assumptions such as the principle where the expired gases are believed to reflect metabolism in active muscles.

The studies on the BL energy equivalent during isolated RE are limited to bench press, leg press and biceps curl exercises (Scott, 2006; Scott et al., 2009; 2011). Scott et al. (2009) verified that in bench press anaerobic energy was predominant in men but that in women it was almost equivalent to that released from aerobic sources (50% 1-RM load). The anaerobic EC (average of men and women) was 3,2 kcal·min^−1^. In a previous study (Scott, 2006) the author separated the anaerobic EC between genders when performing the same exercise at 60 and 80% 1-RM, and verified higher mean values in men (7 and 9 kcal·min^−1^, respectively), when compared with women (1 to 2 kcal·min^−1^). Recently, Scott et al (2011) have confirmed the anaerobic predominance from 37 to 90% 1-RM when bench pressing. Curiously, in the 2006 study (Scott, 2006) anaerobic energy release was minor (20 a 40%) compared to the aerobic fraction in the three exercises above mentioned (at 60 to 80% 1-RM loads in both genders). Anaerobic fraction in biceps curl was 3 kcal·min^−1^ at 60% and it was 1 kcal·min^−1^ at 80% 1-RM in women; and it was 6 kcal·min^−1^ at 60% and 3 kcal·min^−1^ at 80% 1-RM in men. Finally, in leg press anaerobic EC was up to 3 kcal·min^−1^ in women and it was 9 to 10 kcal·min^−1^ (also at 60% and 80% in men). It is worthwhile to mention that none of these estimations added the contribution of the alactic energy to the anaerobic energy cost.

Using the AOD method to assess the EC in RE, our data with trained men suggest that in bench press, triceps extension and latt pull down the anaerobic EC is 7 to 10 kcal·min^−1^, representing from 65 to 80% of total energy release (unpublished data). In the ½ squat at the same relative intensity, we found a mean anaerobic fraction ≈80% with an EC from anaerobic sources up to 36 kcal·min^−1^. According to our results, the AOD during RE may attain values close to 50 ml· kg^−1^min^−1^ in a 30 s duration, which represents a rate of anaerobic energy release higher than that described for maximal intensity running or cycling.

In summary it seems to be quite clear that it is necessary to rely on expired O_2_ measurements to assess the aerobic EC during RE. However, further research is warranted to improve the interpretation of this measure (i.e. further research on O_2_ on-kinetics as a function of exercise intensity, duration or movement speed). The post-exercise VO_2_ measurement may be of interest only in the case of comparative studies and when full sessions of ST are to be analyzed, but not in the bioenergetics characterization of isolated RE; since this measure includes both aerobic and anaerobic metabolisms and it also involves homeostasis mechanisms which do not reflect quantitatively the energy demand during exercise.

Regarding the estimation of the lactic anaerobic EC, it is our opinion that more studies are warranted using both the BL energy equivalent and the AOD. However, these should be, in a first stage, mainly methodological; analyzing possible variations of each method to unravel which of the two is more likely to be valid and precise in the case of RE. The alactic energy release may be investigated though multi exponential modeling of the O_2_ on-kinetics (O_2_ deficit) and off-kinetics (O_2_ debt). However, this approach ought to be complemented with concomitant studies aiming to quantify more precisely the amount of muscle mass involved in each RE exercise (once the alactic energy estimation is dependent on the amount of muscle mass which is active). To date, the available studies show that:

- The EC in RE at the intensities often used in training is predominantly anaerobic.
- The rate of anaerobic energy release in exercises such as squatting may be higher than maximal values described for high-intensity running or cycling.
- The anaerobic EC seem to present different estimations according to the different methods (lactate energy equivalent *vs*. AOD). This mismatch may indicate that the lactate energy equivalent from running, cycling and swimming studies may not be applicable to RE.
- The total EC involved in RE may attain values up to 40 kcal·min^−1^ in exercises which elicit a large muscle mass.

From the previous analysis it is concluded that knowledge about the true energy cost of resistance exercise is still in its early days and much research is still warranted before reliable reference values are available. The large variability in strength training methods and in the execution of resistance exercises (speed, range of movement, type of contraction, etc) implies a wide range of possible studies. However, the next step in the evolution of the state-of-the-art should comprise methodological analysis able to demonstrate which methods, techniques and procedures are to be recommended in this line of research.
